# Variations in seismic parameters for the earthquakes during loading and unloading periods in the Three Gorges Reservoir area

**DOI:** 10.1038/s41598-022-15362-9

**Published:** 2022-07-02

**Authors:** Lifen Zhang, Wulin Liao, Zhigao Chen, Jinggang Li, Yunsheng Yao, Guangqin Tong, Yannan Zhao, Ziyan Zhou

**Affiliations:** 1 Key Laboratory of Earthquake Geodesy, Institute of Seismology, China Earthquake Administration, Wuhan, 430071 China; 2 Institute of Disaster Prevention, Sanhe, 065201 Hebei China; 3 China Three Gorges Corp, Yichang, 443000 China

**Keywords:** Natural hazards, Geophysics, Seismology

## Abstract

As the largest water conservancy and hydropower project in China, the Three Gorges Reservoir is a weak seismic activity area before impoundment, but the frequency of earthquakes increases significantly after impoundment. The spatial density scanning method was used to obtain the characteristics of spatio-temporal earthquake distribution in the reservoir area during loading and unloading processes. The results show that the frequencies of earthquakes during the loading and unloading processes were higher than that during the low-water-level operation period, which is well explained by the acoustic emission test results. The seismic b-value, fractal dimension D, and spatial correlation length SCL can be used together to indicate stress criticality. To analyze the impacts of reservoir water loading and unloading on seismicity in the reservoir area, time-scan analyses were performed on the b-value, D-value, and SCL of earthquakes near the Zigui segment and the Badong segment. Previous studies argued that the time-varying characteristics of b-values do not hold predictive significance for earthquakes in the M4.0–6.2 range. However, our study found that the time-varying characteristics of b-values are of predictive significance for earthquakes around M4.0. These seismic parameters decrease significantly before moderate earthquakes but at different rates in different regions.

## Introduction

With the frequent occurrence of human activities, the problem of induced earthquakes has attracted more attention^1^. Especially in recent years, induced earthquakes have been mostly associated with the development of new energy sources such as shale gas, the exploration of geothermal energy, the construction of gas storage facilities, and mining^2,3^. Reservoir-induced earthquakes as a kind of induced earthquakes have attracted widespread concern in the early stage^4,5^. The construction of large-scale water conservancy and hydropower projects and periodic water impoundment affect the stability of the regional crust and sometimes trigger seismic activity. Since the 1950s, reservoir-induced earthquakes have been studied extensively. The earliest recorded reservoir-induced earthquake was the Lake Mead earthquake in 1945^4^. In the 1960s, four reservoir-induced earthquakes of M ≥ 6.0 were reported^5,6^. Over 140 reservoir-induced earthquakes have been reported worldwide since the 1960s, none of which were above 6 in magnitude.

The occurrence of reservoir-induced earthquakes is related to many factors, such as regional geological structure, permeability of rock mass and tectonic fractures, regional stress fields, and water level change^4–6^. Among these factors, water load and pore pressure effects are the two main causative factors responsible for the induced earthquakes^7,8^. The impact of water on earthquakes in reservoir areas can be classified into the following two situations^9^. (1) There is a direct hydraulic connection between water and the fault. In this case, the water loading increases the pore pressure on the fault, resulting in a decrease in the effective stress^7,8^. In addition, the softening and weakening effect of water reduces the coefficient of friction on the fault plane and reduces the cohesive force, thereby causing earthquakes^10,11^. (2) There is no direct hydraulic connection between water and the fault. Reservoir impoundment or discharge causes changes in the local stress field below and around the reservoir and changes in the fault stress field, thereby inducing earthquakes^2,9^. Previous studies have also investigated the relationship between reservoir water level and seismicity^12–15^. Simpson et al.^12^ summarized that rate changes of reservoir water level is a significant factor in determining the reservoir-induced seismicity. Telesca et al.^13,14^ applied singular spectrum analysis to study the relationship between local seismicity and water level. The results showed that earthquake frequency is related to quasi periodic variation in the water level. Smirnov et al.^15^ pointed out that the amplitudes of the seasonal peaks of the induced earthquakes are not constant but vary significantly with time.

Three Gorges Reservoir is the largest water conservancy and hydropower project in China. Before reservoir impoundment, the reservoir area was characterized by weak seismicity. After reservoir impoundment, the frequency of earthquakes increased significantly. To ensure the safety of the reservoir area during earthquakes, a seismic monitoring network was built in 2001. The network has been in operation for more than 20 years. The data collected covered the whole time period before and after reservoir impoundment. The Three Gorges reservoir provides a natural experimental field and a good opportunity for studying reservoir-induced earthquakes. Some previous studies have focused on seismicity in the Three Gorges reservoir area. For example, Jiang et al.^16^ applied the epidemic-type aftershock sequence model to study the effect of reservoir water loading on earthquakes and concluded that the seismicity was stronger during the rapid loading stage than in the unloading stage. Zhang et al.^17^ conducted correlation analysis and impulse response analysis on earthquake and water level data from 2003 to 2009 and revealed the characteristics of rapid and delayed seismic responses to reservoir impoundment in the surrounding area. Zhang et al.^18^ analyzed the characteristics of the focal mechanism solution of the seismicity near the Fairy Mount fault in the Zigui area during the loading and unloading stages and discussed the seismogenic mechanism of the earthquakes in this region. Since 2008, the Three Gorges Reservoir has entered a periodic loading and unloading stage, and the reservoir water level fluctuates periodically between 145 and 175 m each year. Prior to September 2008, the seismicity in the reservoir area was dominated by micro-small earthquakes, and no earthquakes above M4.0 occurred. However, since 2008, earthquakes in the reservoir area have been more frequent than before.

What are the effects of the periodic impoundment and discharge processes of the Three Gorges reservoir on seismicity in the reservoir area? Are there differences in the characteristics of seismicity at different stages? If the characteristics differ, what is the cause? Based on these questions, this paper discusses seismic responses in the reservoir area to periodic loading and unloading stages from the perspectives of statistical seismology and rock mechanics experiments and analyzes the possible causes. It is very significant for promoting research on the seismogenic mechanisms of reservoir induced earthquakes and determination of seismic trends in reservoir areas.

## Geological structure of the study area

**Figure 1:**
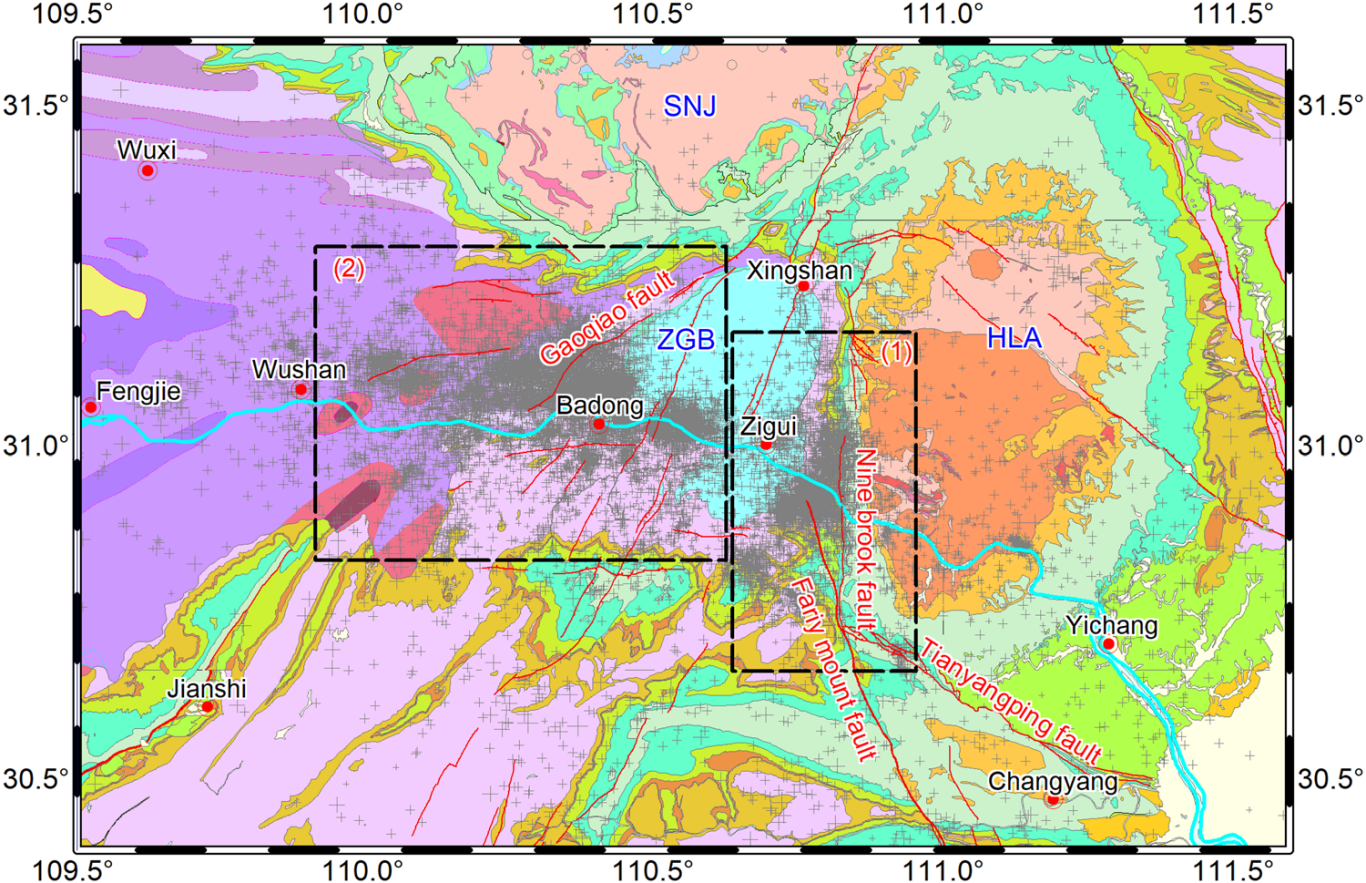
Geological structural map of the head area of the Three Gorges Reservoir. Huangling anticline (HLA), Zigui basin (ZGB), and Shennongjia block (SNJ) are the main tectonic units in the Three Gorges Reservoir area. The black dashed boxes represent the Badong earthquake swarm and the Zigui earthquake swarms, the gray dots represent the distribution of earthquakes since 2003, and red lines represent faults. The map was created using the free software GMT (http://mirrors.ustc.edu.cn/gmt/).

The Three Gorges reservoir is located in the upper reaches of the Yangtze River. The dam is built on top of the granite on the southeastern margin of the Huangling dome. The reservoir is controlled by two tectonic units, the Huangling dome and the Zigui basin. The Pre-Sinian crystalline basement and the Sinian–Cretaceous strata outcrop at the core and two wings of the Huangling dome. Cretaceous glutenite strata are distributed in the Fairy Mount fault zone to the southwest of the Huangling dome. Limestone and karst are widely distributed along the Gaoqiao fault at the western margin of the Zigui Basin and in the regions downstream of Fengjie^19^ (Fig. 1).

The faults that cross the reservoir include the Fairy Mount fault, the Nine-brook fault, the Tianyangping fault, the Yuanan fault, the Gaoqiao fault, and the Xinhua fault (Fig. 1). (1) Fairy Mount fault is a NNW-trending fault, which is located on the southwest side of the Huangling dome, 19 km from the dam site. The fault dips west at 60°–70° and is nearly 100 km long. The fault zone is mainly composed of breccia and cataclastic rocks, showing dextral shear compression. (2) Nine-brook fault is a nearly NS-trending fault and is located on the southwest side of the Huangling anticline, approximately 17 km from the dam site. The fault dips west at 50°–60° and shows tensional shear faulting. (3) Tianyangping fault is a NW-trending fault, which is located on the north wing of the Changyang anticline, with a total length of 60 km. Its west segment is cut off by the Fairy Mount fault. The Fairy Mount fault, the Nine-brook fault, and the Tianyangping fault converge and form a K shape. (4) Gaoqiao fault is a NE-trending fault on the western margin of the Zigui Basin. It shows compressive shear faulting. (5) Xinhua fault is located between the Shennongjia fault dome and the Huangling anticline, with a total length of 50 km. It consists of breccia, broken silt, and well-developed fault gouge.

## Data and methods

### Data

**Figure 2:**
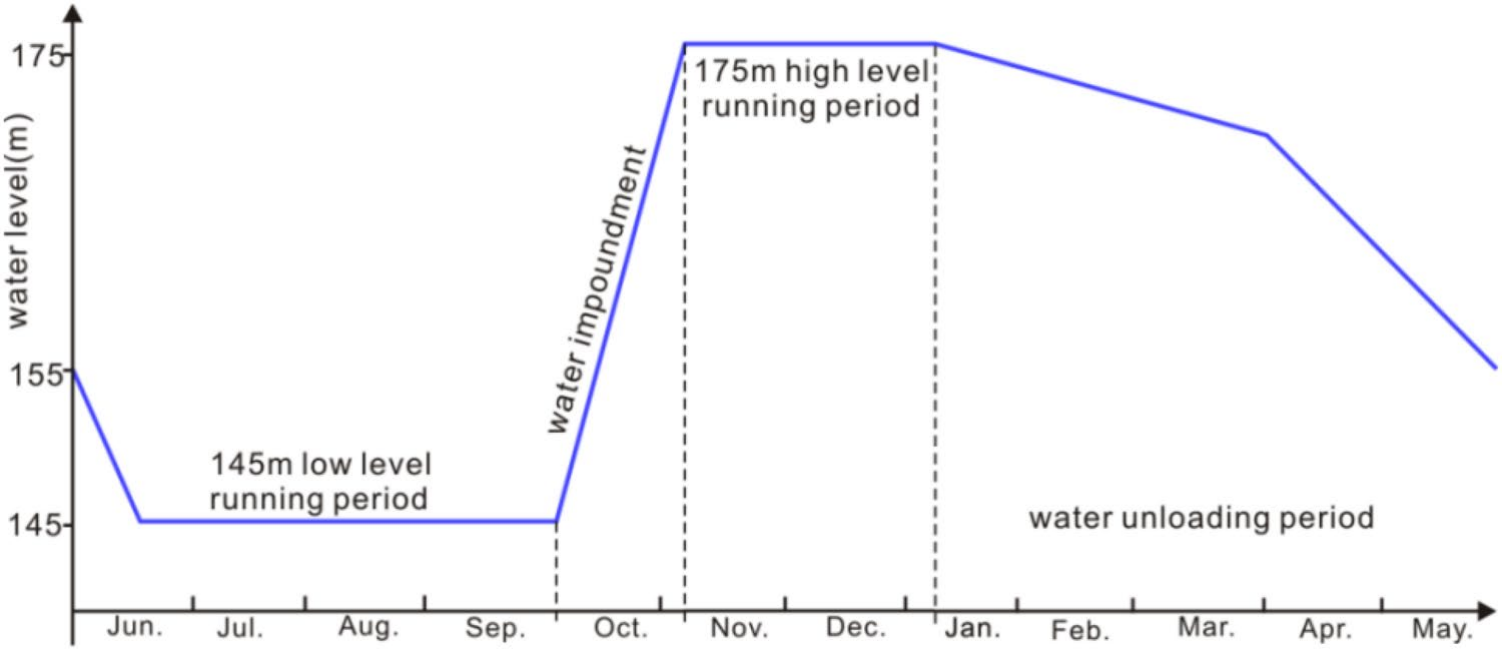
Reservoir water level planning curve in the Three Gorges reservoir.

**Figure 3:**
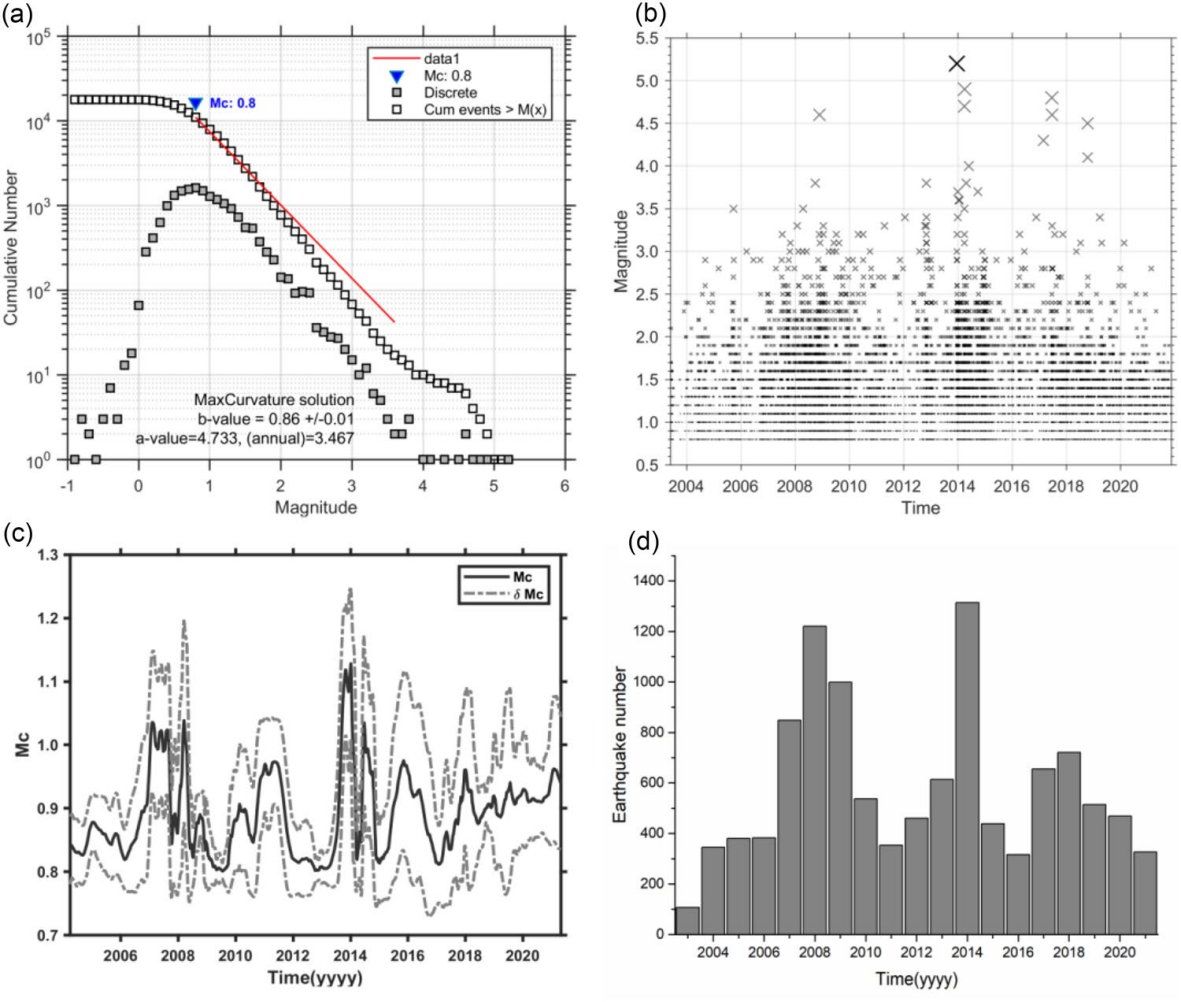
(**a**) Scatter plot of magnitude of events against the frequency indicates the completeness of all the earthquakes (M_c_) as 0.8; (**b**) Magnitude with time; (**c**) Mc varies with time, dashed black lines are the standard error of Mc; (**d**) Yearly frequency of earthquakes with time.

In 2001, the digital network for monitoring reservoir-induced earthquakes in the Three Gorges reservoir was completed and put into operation, consisting of 24 seismic stations. In 2012, the network was reconstructed, and was composed of 22 seismic stations. The Three Gorges reservoir began to impound in 2003 and entered a periodic water storage stage in 2008. The period from January to May is the period of reservoir water unloading, when the water level drops from 175 to 145 m. The period from June to August is the flood season, which is the stage for saving the reservoir storage capacity, during which the water level remains unchanged at 145 m. From September to December, the reservoir operates at high water levels, and the water level increased from 145 to 175 m (Fig. 2). This paper selects the reservoir water level and seismic data from June 2003 to April 2020 for analysis. Since the first impoundment of the reservoir, over 10,000 earthquakes have been recorded in the reservoir area. 92% of the earthquakes were M0.0–1.9 microearthquakes, and the remainder were small and moderate earthquakes (Fig. 3b,d). There were 788 M2.0–2.9 earthquakes, 71 M3.0–3.9 earthquakes, and 8 M4.0–4.9 earthquakes. The largest earthquake was the M5.1 earthquake in Badong on December 16, 2013. To ensure completeness of seismic data sets, complete magnitude (Mc) is estimated using ZMAP software^20^. As shown in Fig. 3, Mc is 0.8 (Fig. 3a,c).

## Research methods

1. ***b*-value**

Gutenberg and Richter^21^ proposed the Gutenberg–Richter relation that represents the distribution of earthquake magnitude and frequency: log *N* = *a-bM*. The coefficient *a* is mainly determined by the maximum earthquake magnitude in the sequence. The b-value is a function of the relative earthquake magnitude distribution and is an important parameter for measuring the level of seismicity, which reflects the stress state of the rocks^22^. Time variations in *b*-value with loading stress and time can reflect the initiation, propagation, and instability of rock cracks, which is of important physical significance. There are two main methods for estimating the *b*-value: the least-squares method and the maximum likelihood method^23^. The least-squares method linearly fits the *M*-log*N* relationship to obtain the *b*-value. The maximum likelihood method is based on the earthquake probability density function and applies the following formula to calculate the *b*-value^20^:

1 $$b = \frac{\log e}{{\overline{M} - M_{c} }}$$

The standard error of *b* is estimated using the formula^20^:

2 $$\sigma \left( b \right) = 2.3b^{2} \sqrt {\sum\limits_{i = 1}^{n} {\frac{{\left( {M_{i} - \overline{M}} \right)^{2} }}{{n\left( {n - 1} \right)}}} }$$

Here, $\overline{M}$ represents the average magnitude of the earthquake, *n* is the number of the earthquakes, and *M*_*c*_ represents the magnitude of completeness. According to previous studies, the maximum likelihood method is preferred in the practical calculation of the *b*-value. Because the maximum likelihood method provides an unbiased estimation based on simpler calculation, the results are more stable. However, the two methods are not stable if the sample size is small. In the calculation, it is necessary to select as many samples as possible and a small value of *M*_*c*_.

2. **Fractal dimension** ***D*** **and spatial correlation length (SCL)**

Earthquakes are usually distributed in clusters, which can be quantified using fractal dimension values^24^. If the hypocenter distribution possesses fractal characteristics, then

3 $$C\left( r \right) = \frac{{2N_{r} \left( {R < r} \right)}}{{N\left( {N - 1} \right)}}$$

*N*_*r*_((*N*) < *r*) is the number of seismic hypocenter pairs separated by distances less than *r*, and *N* is the total number of seismic events. If the hypocenter distribution shows fractal characteristics, then *C*_*q*_(*r*) is a power function of *r*, i.e*.*, $C_{q} \left( r \right)\infty \;r^{{D_{2} }}$*.* Here, *D*_2_ defines the correlation dimension^23^. In addition to the fractal dimension *D*, SCL can also be used to characterize the spatial distribution of hypocenters. Single-link cluster analysis is used to estimate the SCL of *N* consecutive events^23^. Initially, each independent hypocenter is connected to the nearest hypocenter to form a group of clusters. Then, each earthquake cluster is connected to its nearest earthquake cluster. This process is repeated until *N* events are connected to *N*-1 links^25^. In the entire process, the distance between any two clusters is calculated based on their geometric centers. According to previous studies^25^, SCL is defined as the median of the length distribution of *N*-1 links.

## Results

### Spatial variations in earthquakes in the reservoir area

**Figure 4:**
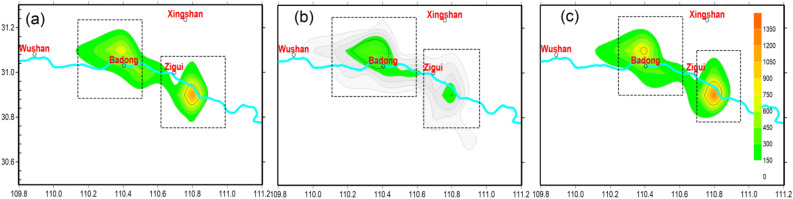
Contour map of the number of events per month from September 2008 to April 2020 during different time periods. (**a**) From January to May, (**b**) from June to August, (**c**) from September to December.

To study the evolution of earthquakes during the periodic process of water loading and unloading, the spatial scanning method is used to analyze the characteristics of earthquake frequency variation. Figure 3 shows the spatial variations in the monthly frequency of earthquakes in the head area of the Three Gorges reservoir during loading and unloading periods, with a seismic scanning step of 0.1°. Spatially, earthquakes are mainly distributed along the Yangtze River, generally near the Zigui segment of the Fairy Mount fault and the Badong segment of the Gaoqiao fault within 10 km of both banks of the reservoir. Except Zigui and Badong area, a very small number of earthquakes scattered in some other regions. Therefore, in this study, only the earthquakes in the two regions are analyzed and discussed. During the two periods from January to May and from September to December, the overall frequency of earthquakes near the Fairy Mount fault was slightly higher than that in the Badong area. Whereas, during the period from June to August when the reservoir operated at a low water level of 145 m, the frequency of earthquakes in the Badong area was slightly higher than that in the Zigui area (Fig. 4).

Spatially, the seismicity in the Three Gorges reservoir area is consistent with the common features of reservoir-induced earthquakes^5^. Generally, the reservoir induced earthquakes are basically limited within 10 km of each bank of the reservoir, which is determined by the impact range of the water loading on the reservoir area and the limited seepage range of the reservoir area^5,9^. This limited range corresponds to the main area of adjustment of the tectonic stress field^5^. In addition, reservoir-induced earthquakes often occur near the intersections of faults, karst cave development areas, and contact zones with different lithologies. The intersection of the NW-trending Fairy Mount fault with the nearly NS-trending Nine-brook fault and the north end of the Fairy Mount fault are stress-concentrated regions, where the earthquakes are clustered. Moreover, multiple sets of joint fissures are developed in these areas, which have direct hydraulic relationships with reservoir water. A karst conduit system is developing in the Badong area, and Triassic weak detachment layers are developing in the area, which are conducive to the occurrence of reservoir-induced earthquakes^26^.

### Characteristics of temporal variation in earthquake frequency

**Figure 5:**
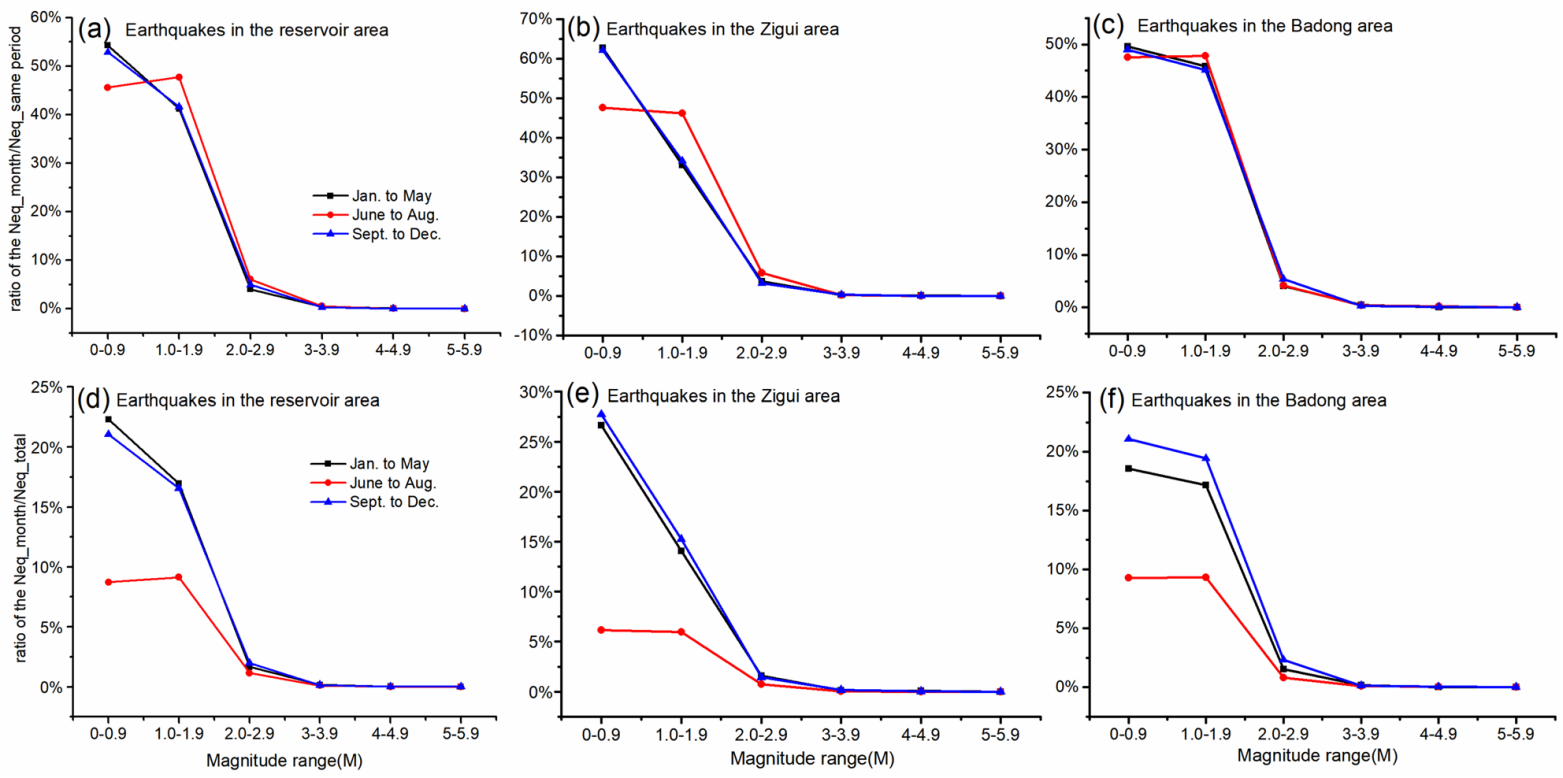
Monthly proportions of earthquakes of different magnitudes in the Three Gorges Reservoir area and the Zigui and Badong subregions at different loading and unloading stages.

We analyzed the time variations in magnitude and frequency of the earthquakes in the entire head area of the Three Gorges reservoir and the above two subregions. During the water loading period from September to December, the unloading period from January to May, and the low-water-level operation period from June to August, more than 90% of the earhthquakes are micro-earthquakes below M2.0. The proportions (*a*_*mj*_) of earthquakes with same magnitudes to the total number of earthquakes in the corresponding three periods follow a descending trending with magnitude increases (Fig. 5a–c). The ratio (*a*_*mj*_) of *mj* = 0.0–0.9 during the low-water-leve operation period from June to August is less than the other two stages, especially for the Zigui area (labeled with red solid line in Fig. 4a,b). Whereas, the ratio of *mj* = 1.0–1.9 during the low-water-leve operation period is larger than the other two stages (Fig. 4a,b). For the other magnitude ranges, the differences of the ratios in the three stages are very small. The blue and black solid line representing the ratios for the water loading and unloading periods almost duplicated, which denotes that the impact of water loading and unloading on seismicity are identical.

4 $$a_{mj} = \frac{{\sum\limits_{i}^{{}} {\left( {N_{mj} } \right)_{i} } }}{{\sum\limits_{i}^{{}} {\left( {\sum {N_{mj} } } \right)_{i} } }} \times 100\% ,\;\;\left( \begin{gathered} mj = 0.0 - 0.9,1.0 - 1.9,2.0 - 2.9, \\ 3.0 - 3.9,4.0 - 4.9,5.0 - 5.9 \\ \end{gathered} \right)$$

5 $$b_{mj} = \frac{{\sum\limits_{i}^{{}} {\left( {N_{mj} } \right)_{i} } }}{{\sum\limits_{i = 1}^{12} {\left( {\sum {N_{mj} } } \right)_{i} } }} \times 100\% ,\;\;\left( \begin{gathered} mj = 0.0 - 0.9,1.0 - 1.9,2.0 - 2.9, \\ 3.0 - 3.9,4.0 - 4.9,5.0 - 5.9 \\ \end{gathered} \right)$$

where *N*_*mj*_ is the monthly frequency of earthquakes with a magnitude of *mj*, *mj* is the earthquake magnitude, and *i* (from 1 to 5, from 6 to 8 and from 9 to 12) shows the unloading period, the low-water-level period and water loading period, respectively.

**Figure 6:**
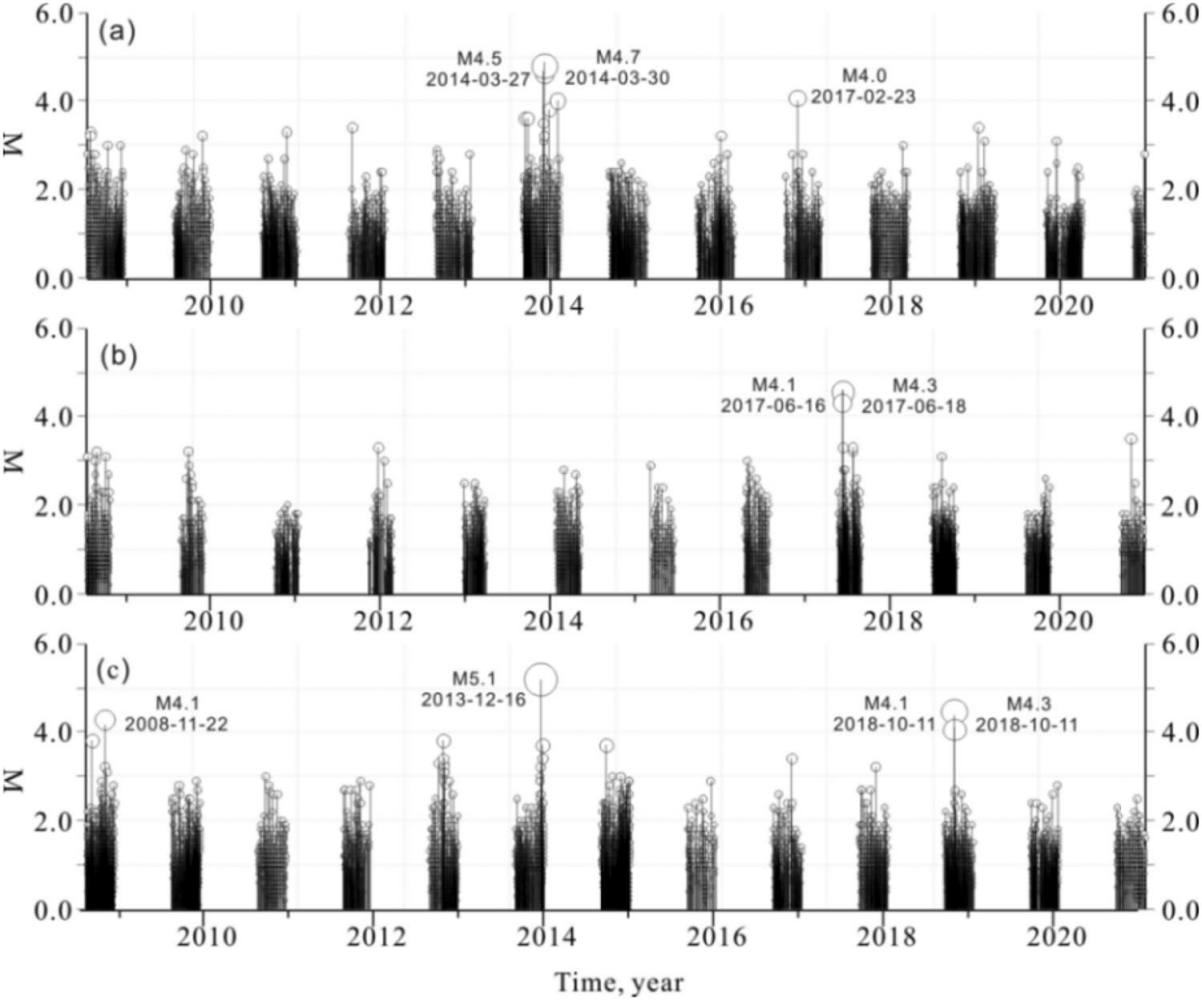
Characteristics of variation in earthquake magnitude in the reservoir area in different reservoir impoundment and discharge periods.

The proportions of earthquakes in the three periods out of the total number of earthquakes (*b*_*mj*_) were calculated (Fig. 5d–f), and the frequency of earthquakes in the loading and unloading stages was significantly higher than that in the low-water-level operation period. The numbers of micro-earthquakes below M2.0 in the loading and unloading stages in the Zigui area were generally 3 to 5 times the number of earthquakes in the low-water-level operation period, and the numbers of micro-earthquakes below M2.0 in the loading and unloading stages in the Badong area were approximately twice the number of earthquakes in the low-water-level operation period. During the process of reservoir water unloading from January to May, 3 earthquakes above M4.0 occurred in the Zigui area. During the low-water-level of 145 m operation period from June to August, 2 earthquakes above M4.0 occurred in the Badong area. During the loading process from September to December, a total of 4 earthquakes above M4.0 occurred in the reservoir area (Fig. 6). The impacts of reservoir water loading and unloading on seismicity in the reservoir area was mainly shown in the differences in micro-earthquakes and small earthquakes.

### Variations in b-value, fractal dimension value D, and SCL over time

The seismic *b*-value, fractal dimension *D*, and SCL can be used together to indicate stress criticality^24^. In general, the *b*-value is a function of rock properties and stress level^24^. If the earthquake distribution shows fractal characteristics, then *D* defines the correlation dimension, which is important for the prediction of seismic hazard^27^. SCL also can be an indicator of the critical state of earthquake nucleation. Time-scan analysis is performed on the *b*-value, *D*, and SCL of earthquakes in the Zigui and the Badong area. The scanning period was from June 2003 to April 2020 and the statistical parameters were estimated for consecutive groups of 200 events with a running step of 50 events.

**Figure 7:**
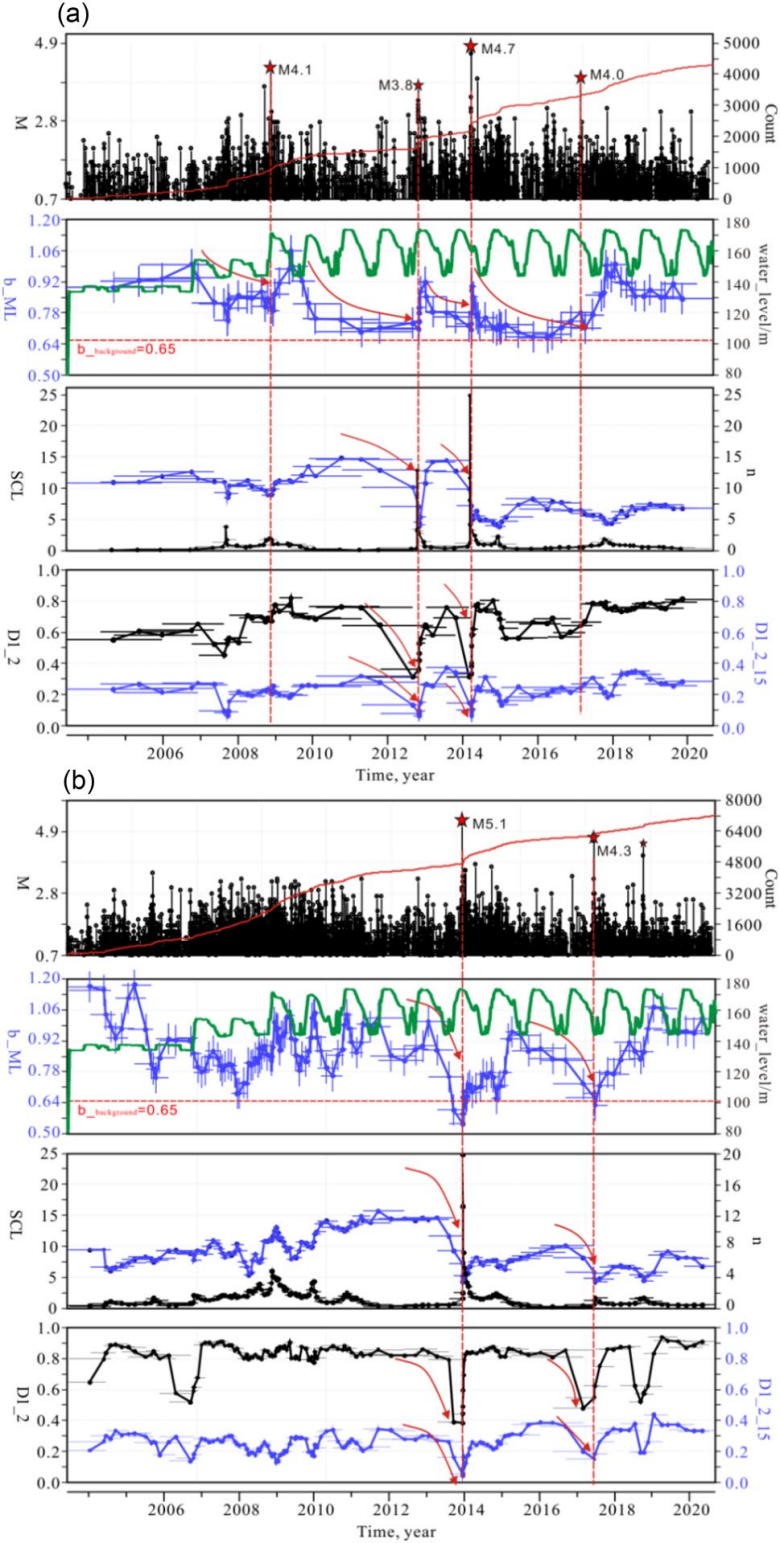
Magnitude, cumulative event number and various statistical parameters (e.g. *b*-value, D and spatial correlation length SCL) estimated for M0.8 + earthquakes from 2003 to 2020. The parameters were estimated for consecutive groups of 300 events with a running step of 50 events. Red vertical dashed lines show the positions of the M4.0 + earthquakes in Zigui and Badong area. The vertical blue bars in *b*_value show the standard error and the horizontal bars in *b*_value, D and SCL show the time scan window. Red horizontal line shows the background *b*-value in the Three Gorges reservoir. The red arrow lines indicate the trend of the statistical parameters. Green solid line shows the water level from May 2003 to May 2020 Red stars indicate the moderate earthquakes with M ≥ 4.0.

Figure 7 shows the time variations in *b*-value, *D*, and SCL in the two areas. Figure 7a shows the *b*-value variations of earthquakes in the Zigui area. The *b*-value slowly decreased before moderate earthquakes and rapidly recovered after the main shocks. An M4.1 earthquake occurred on November 22, 2008, with a *b*-value of approximately 0.8. After the occurrence of the main shock, the *b*-value rapidly increased to approximately 1.0. After April 2009, the *b*-value began to gradually decrease. On October 31, 2012, the *b*-value decreased to 0.68, and an M3.2 earthquake sequence occurred in the Zigui area. After that, the *b*-value recovered rapidly and then decreased slowly. When the *b*-value decreased to the local minimum in March 2014, the M4.5 and M4.7 earthquakes occurred in the Zigui area. After the main shock, the *b*-value increased rapidly and then decreased slowly. In February 2017, an M4.0 earthquake occurred in the Zigui area. Afterward, the *b*-value continued to return to high values and continued to the present, during which no earthquakes above M4.0 occurred.

The *b*-values of the earthquakes in the Badong area also decreased rapidly before moderate earthquakes but recovered relatively slowly afterward (Fig. 7b). In 2003, many micro-small earthquakes instantly occurred due to water impoundment. The *b*-value during the period exceeded 1.0, which is a typical feature of reservoir induced earthquakes^5^. Later, *b*-values varied with water level changes. Since the water impoundment in September 2008, the *b*-value in this area has changed slowly, with minor local variations. In December (high-water-level operation period) 2013, when the water level reached 175 m, the *b*-value suddenly decreased to the background *b*-value (0.65). The time interval for the sudden change in the *b*-value was 12 months, and then the *b*-value slowly returned to the level before the main shock. The *b*-value started to decrease from January 2015, and the M4.3 and M4.1 earthquakes occurred in June 2017. Afterward, the *b*-value slowly recovered. An M4.1 earthquake occurred again in October 2018 when the *b*-value declined. In October 2018, the *b*-value recovered to a relatively high level and continued to the present, and the frequency of earthquakes has been significantly lower than in the past. The variations in SCL and *D* values were generally consistent with the trend in *b*-value variation, decreasing before the occurrence of a moderate earthquake and recovering after the main shock (Fig. 7b).

## Discussion

1. **Characteristics of reservoir induced seismicity during different periods.**

Previous studies show that reservoir induced seismicity can be classified into two types: rapid response and delayed response^28^. For the former type, the earthquakes are significantly correlated with water impoundment. Whereas, the relationship between earthquakes and water level changes is more complex during the late stage of impoundment^29^. In the case of the seismicity in the Three Gorges reservoir area as mentioned above, similar characteristics are shown. As a whole, the frequency of earthquakes during the loading and unloading periods was significantly higher than that during the low-water-level operation period (Figs. 4, 5).

**Figure 8:**
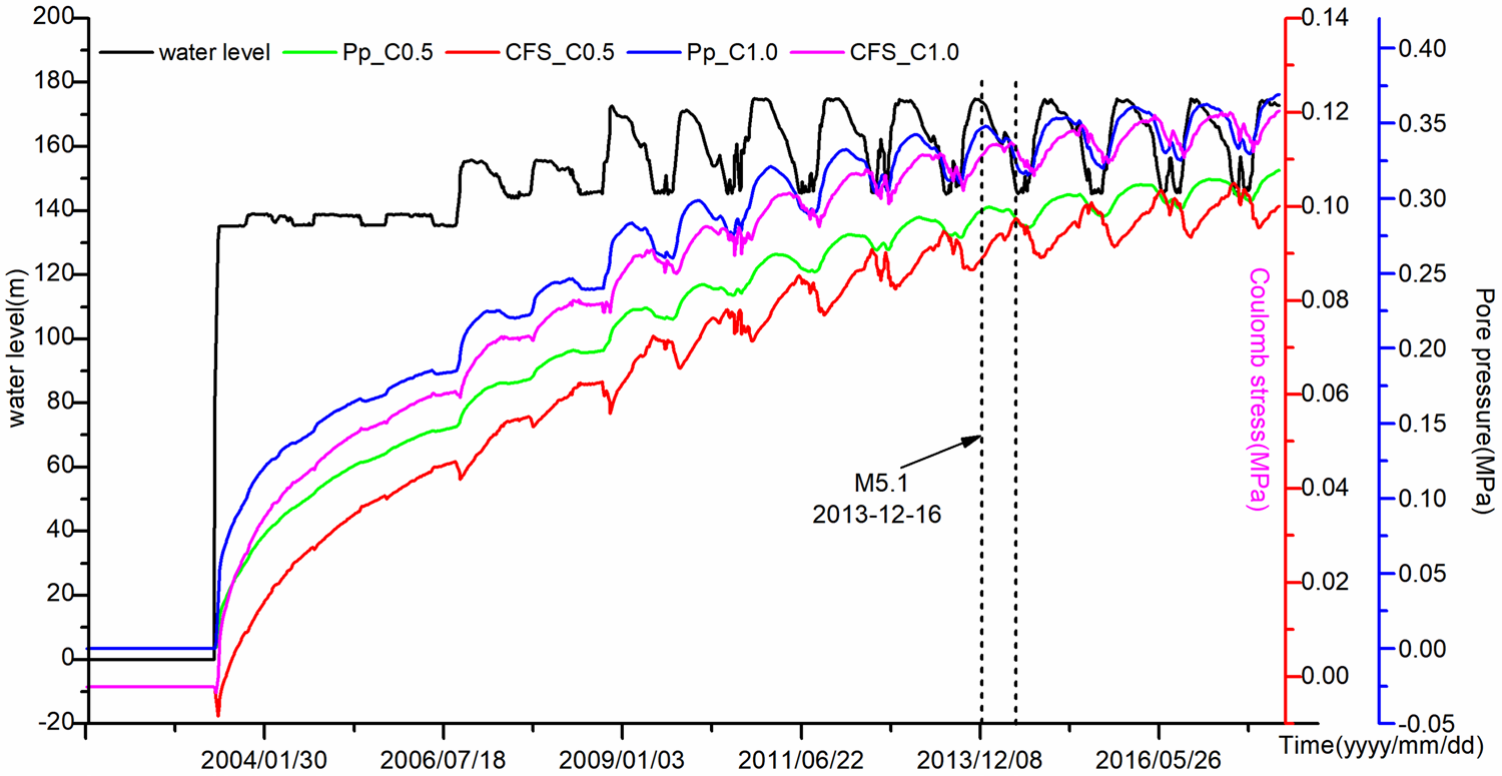
Water level changes (black solid line), diffused pore pressures (green and blue lines) and coulomb stress (red and pink lines) associated with M5.1 earthquake on 16, December, 2013 for different hydraulic conductivity c.

Referring to the rock acoustic emission experiment results^30,31^, we explored the possible reasons. In the early stage of reservoir impoundment, with the increasing of the water level, some karst caves or abandoned mine caverns were flooded with water, resulting in frequent occurrences of collapse-related micro-earthquakes and small earthquakes^32^. These earthquakes could be categorized as instantaneous response of reservoir water impoundment and mainly clustered in the Badong area. With increasing water loading stress, reservoir water diffuses horizontally and penetrates along the fractures, and the increase in pore pressure is conducive to the propagation and coalescence of cracks^11,29^. Crack propagation and coalescence in turn promotes fluid pressure diffusion, resulting in a decrease in the effective stress of the fault^28,29^. The superposition of fluid pressure diffusion and water loading stress controls the coulomb stress of a fault. When the critical stress is reached, the fault slips due to instability. At this stage, moderate earthquakes are likely to occur. It is known that fluid pressure diffusion is a relatively slow process, therefore, there is a time-delay between water impoundment and earthquakes^5,10^ (Fig. 8).

Figure 8 shows the time variations of pore pressure and coulomb stress at the source area of the Badong M5.1 earthquake on 16, December 2013 with different hydraulic conductivity coefficients. From May 2003 to September 2006, the reservoir water level rose rapidly from 80 to 135 m, and then fluctuated between 135 and 145 m. During this period, the pore pressure and coulomb stress at the source increase exponentially. When the diffusion coefficient is small, the pore pressure diffusion under undrained effect cannot be ignored; while when the coefficient becomes larger, the pore pressure diffusion under drained effect is much larger than that under undrained pore pressure^10^. Therefore, during the period of initial impoundment, the porosity is very small and the pore pressure of the tight rock mass increases instantaneously due to the undrained effect. In the circumstance, some small earthquakes are induced. Figure 8 shows that the greater the hydraulic conductivity coefficient is, the greater the pore pressure and coulomb stress are. Since September 2006, when the reservoir water level shows a periodic changes, both the pore pressure and coulomb stress also exhibited periodic changes (Fig. 8). A significant time-delay positive correlation exists between the reservoir water level change and stress changes. When the Badong M5.1 earthquake occurred, the coulomb stress at the source area was greater than 0.1 MPa and indicated a high risk at this time. During the high-water-level operation, pore pressure diffusion under drainage effect especially along the fault and large porosity rock mass resulted in the occurrences of moderate earthquakes^26^.

The acoustic emission experiment results showed that there was no acoustic emission event during the period when the loading stress remained unchanged at a low level^30,31^. Correspondingly, the number of the earthquakes in the Three Gorges reservoir area was relatively low during the low-water-level operation period from June to August. However, due to the gradual seepage and diffusion of the reservoir water to the deep, seismicity continued for a long time, but the frequency of earthquakes was lower than that during the loading and unloading periods.

2. **Significant decreases in the** ***b*-value,** ***D***_**2**_**, and SCL are of indicative for the prediction of moderate earthquakes in the Three Gorges reservoir area.**

**Figure 9:**
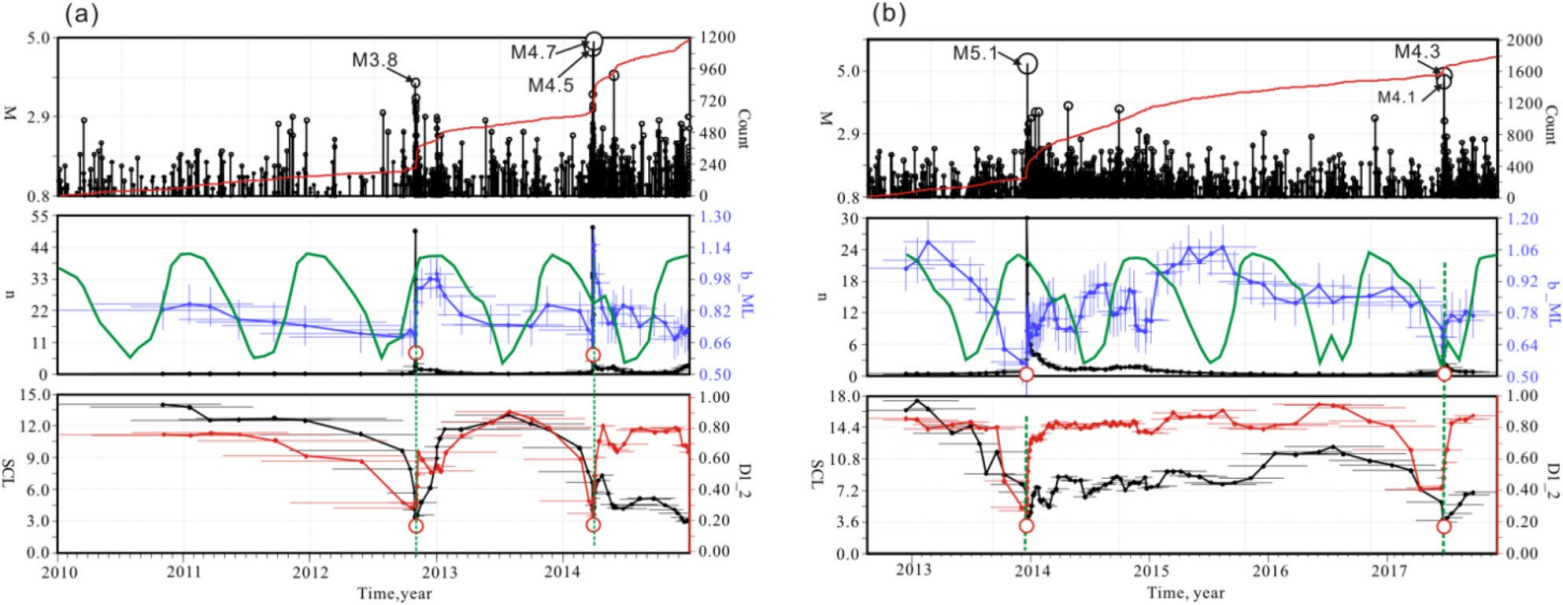
Temporal variations in earthquake accumulated number, magnitude, *b*-value, SCL, and *D* in the Zigui area (**a**) and Badong area (**b**). Green solid lines show the water level variations with time. Black empty circles show the moderate earthquakes occurred in the Zigui and Badong area.

Parsons et al.^33^ argued that the time-varying characteristics of *b*-value do not hold predictive significance for earthquakes in the M4.0–6.2 range. However, our study show opposite opinion that the parameters such as *b*-values in the Badong and Zigui areas all exhibited significant decreases before moderate-magnitude earthquakes around M4.0 (Fig. 9). The M4.5 and M4.7 earthquakes occurred in March 2014, when *b*-values decreased to the local minimum. After the main shock, the *b*-values quickly recovered and rapidly entered a state of decrease. In February 2017, an M4.0 earthquake occurred in the Zigui area (Fig. 9a). Afterward, *b* continued to return to high values and remained high to the present, during which no earthquakes above M4.0 occurred. In the Badong area, there was also a significant decrease in *b* before moderate-magnitude earthquakes. During the high-water-level (175 m) operation period in December 2013, the b-value suddenly decreased to the level of the regional background *b*-value. The largest earthquake since the first impoundment of the reservoir area occurred. Afterward, the *b*-value returned to the level before the main shock. When the *b*-value decreased to the level of background *b*-value again in June 2017, an M4.3 earthquake occurred (Fig. 9b). The study of Nuannin^34^ also supports our finding that the *b*-value is indicative of the prediction of moderate-magnitude earthquakes. Gupta^35^ also pointed out that some precursory changes in *b*-value, *D*, stress drop and corner frequency have been noticed prior to moderate earthquakes in the Koyna-warna reservoir area. With the development of seismic observations, more and more researches reveal that the decrease in the *b*-value can be a precursory before large earthquakes^36^.

The long-term variations in the *b*-value reflect the effect of increasing pore pressure diffusion^37,38^. For a given region, a decreased *b*-value can be a good indicator for stress increase^39^ or pore pressure diffusion^40,41^. In the servo control test of the whole rock fracture process, the *b*-value decreased with increasing stress before the fracture and decreased significantly when the rock was close to fracture^42^. After the rock started to fracture, the *b*-value remained low. When the large stress drop stopped and the rock had broken and maintained a substantially constant residual strength, the *b*-value increased again when the dislocation occurred again. The same phenomenon has been observed in the stick–slip process of immature faults^43^. According to the experimental results of Lei et al.^44^, the increases in *D* and SCL reflect the propagation of microcracks, which indicates the nucleation and propagation of faults. The *b*-value, *D*, and SCL decreased rapidly when moderate earthquakes occurred, indicating that the proportion of large-scale microcracks began to increase rapidly^45^. The microfractures inside the rocks began to change from disorder to order. The geometric fractal dimension changed from large to small. When the stress is close to the peak strength, the *b*-value, the fractal dimension *D*, and the SCL decrease to local minima, the clustering of seismic events is obvious, and the cracks in rock start to coalesce and eventually lead to rock instability and failure. Statistical parameters are of great significance for earthquake disaster assessment, and are helpful to understand the evolution process of earthquakes (Smith 1981). In particular, the quantitative analysis of *b*-value changes can provide quantitative information for probabilistic prediction of major earthquakes and are indeed indicative for earthquake trend analysis.

3. **Seismic parameters such as** ***b*-value,** ***D*-value, and SCL-value decrease significantly before moderate earthquakes but at different rates in different regions.**

As mentioned above, seismic parameters before the occurrence of moderate earthquakes in the reservoir area showed significant decreases. However, the parameters decreased at different rates in the two areas. In the vicinity of the Zigui area, the *b*-value decreased slowly before the occurrence of a moderate earthquake, and rapidly recovered within short time after the main shock, and then continued to decrease *D* and SCL also decreased overall, reaching local minima at the time of the main shock, but they decreased more rapidly than the *b*-value. On the other hand, the *b*-value decreased relatively rapidly in the Badong area but recovered more slowly after the earthquake *D* and SCL also exhibited significant time dependence, and both decreased to local minima at the time of the main shock. Rock mechanics experiments confirmed the existence of these two conditions.

The study of Rivière et al.^46^ showed that the rate of change in the *b*-value is closely related to change in normal stress and shear stress. The experimental results show that a fault is in a stick–slip state under high normal stress and shear stress^46^. When the stress increases, the *b*-value decreases till to the local maximum, the *b*-value decreases rapidly. When the fault is in the transitional stage between stick–slip and steady slip, the normal stress and shear stress are slightly lower than in the stick–slip stage, and the *b*-value decreases at a slower rate. In addition, the rate of change in parameters such as *b* may reflect crack propagation speed, which is also closely related to rock properties, the characteristics of the internal rock structure, and the density of the cracks^47^. Rocks with higher strength and brittleness store more elastic energy in the elastic stage^30^. When a rock is about to fail, the stored energy is released intensively, and the reduction in the acoustic emission *b*-value is greater^48^. The difference in regional geological tectonic conditions may be one of the reasons that caused the difference in the rates of change in the *b*-value, *D*, and SCL between the two regions.

## Conclusion

1. There are many similarities between the seismicity characteristics of the Three Gorges reservoir area under periodic water loading and the results of the rock acoustic emission experiment. The spatial density scanning method was used to obtain the characteristics of spatio-temporal earthquake distribution in the reservoir area during loading and unloading processes. The results show that the frequencies of earthquakes during the loading and unloading processes were higher than that during the low-water-level operation period, which is well explained by the acoustic emission experiment results. The impact of reservoir water loading and unloading on seismicity in the reservoir area is mainly exhibited in the differences in microearthquakes and small earthquakes, while the relationship between relatively large earthquakes and reservoir water loading and unloading is not significant.
2. The set of seismic statistical parameters are analyzed using the time-scan method with a fixed number of events. The time dependence of seismic activity in the first area of the Three Gorges Reservoir is analyzed, and the anomaly characteristics before moderate (M4.0 +) earthquakes are distinguished. Before the moderate earthquake, the reservoir area showed a significant decrease in the *b*-value, D and SCL value. This shows that the time variations of the statistical parameters can reflect the characteristics of seismicity, and the critical behavior of the pre-earthquake seismogenic system has important guiding significance for seismic risk analysis. However, at this stage, it is still difficult to rely solely on these statistical parameters to predict earthquakes, and more detailed work needs to be done to distinguish the implicit information unrelated to seismic activity in these parameters. In the future work, we plan to compute the stress drops in loading and unloading processes and analyze the effect of stress drop differences on parameter variations.

## Data Availability

The data sets generated and/or analysed during the study period should not be made publicly available [since the data comes from the seismic network built by the Three Gorges Group Enterprise, the author has the right to use the data but has no public authority], but can be obtained from the corresponding author if reasonable requirements exist.
